# A Phenomenological Perturbation-like Approach for Prediction of Molecular Properties in Large Libraries of Polysubstituted Derivatives: Application to Molecular Solar Thermal Systems

**DOI:** 10.1021/acs.jctc.4c01483

**Published:** 2025-01-08

**Authors:** Alba Peinado, Alejandro Jodra, Claudia Cebrián, Luis Manuel Frutos

**Affiliations:** † Departamento de Química Analítica, Química Física e Ingeniería Química, Universidad de Alcalá, E- 28871 Alcalá de Henares, Madrid, Spain; ‡ Instituto de Investigación Química “Andrés M. del Río”, Universidad de Alcalá, E- 28871 Alcalá de Henares, Madrid, Spain

## Abstract

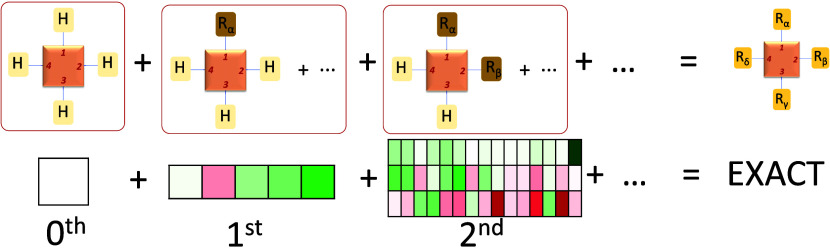

The prediction of a specific chemical property across a vast library of derivatives represents a formidable challenge. Conventional computational methodologies typically rely on brute-force calculations involving the computation of the property of interest for the entire library or a significant subset. In this study, we present a novel phenomenological approach to address this challenge, employing a perturbation theory-like framework to describe substituent effects. This proposed methodology has the potential to forecast the molecular properties of millions of compounds based on information derived from just a few hundred. This method is applied to the design of molecular solar thermal (MOST) systems, which are devices permitting harvesting solar energy and storing it in a chemical form. The optimization of MOST performance is a critical issue in practical applications of this technology, so exploration of large libraries of derivatives at low computational cost is an interesting approach to tackle the problem. To accomplish this objective, we explore the functionalization of the norbornadiene/quadricyclane (NBD/QC) system utilizing the proposed perturbational approach predicting the energy of 350 derivatives from small sets of 5 and 50 calculated compounds.

## Introduction

Molecular design is a central issue in various fields of chemistry. One remarkable example is drug design, where exploring derivatives from a given pharmacophore can be a useful technique.^1^ Using substituents to create new derivatives is also a common strategy in combinatorial chemistry.^2^ This kind of investigation requires the systematic identification of many compounds to find and select the most promising ones. Additionally, by examination of a small set of substituents and diverse substitution sites, the number of resulting compounds can become too large for both computational and experimental analysis. For instance, even a small library of 10 substituents in a system with 7 substitution sites leads to over 10 million possible compounds.

In the past, several empirical formulas have been developed to understand the impact of substituents, especially in relation to the free energy (reaction or activation energy) change. One such noteworthy formulation is the Hammett equation, which links the substituent effect with reaction energy.^3^ The Hammett equation has undergone revisions to account for electronic energy variation caused by the substituent and the corresponding forces that contribute to this variation, which is the origin of the energy change.^4^ Additionally, more advanced methods such as quantitative structure–activity relationship (QSAR) methodologies can establish correlations between a compound’s structure, including its substituents, and its chemical properties.^5^

The development of efficient molecular solar thermal (MOST) systems highlights the importance of molecular design. These systems are molecular entities that can capture solar energy, primarily in the visible range, and store it in a chemical form.^6^ This stored energy can be released in a controlled manner to produce heat when needed.^7^ However, the design of MOST systems is a complex challenge that requires the optimization of various physicochemical properties simultaneously, including absorption energy, reaction energy, activation energy, and photoreaction quantum yield.^8^ Although each property can be studied independently, a holistic approach involves cross-referencing the data to identify the optimal compounds.

A very common strategy to improve MOST performance is to explore derivatives through chemical substitution and study their effects on each individual property of the MOST system.^9^ This can be done by carefully selecting the substituents based on their physical and chemical properties and the expected effect on the different MOST properties,^10^ or by exploring a large range of derivatives in a more systematic way, using for instance a computational brute-force approach.^11^

In any case, the effect of the substituent can be complex and affect many MOST properties in very different ways, due to the electronic effect of the substituent,^12^ steric effects that can eventually be understood as mechanical forces.^13,14^

The norbornadiene/quadricyclane (NBD/QC) MOST system is a reference molecular architecture designed for efficient solar energy capture and storage.^15^ This system is characterized by its photochemical behavior, primarily involving the reversible isomerization between the closed-ring isomer, quadricyclane (QC), and the open-ring isomer, norbornadiene (NBD).^16^

**Figure 1:**
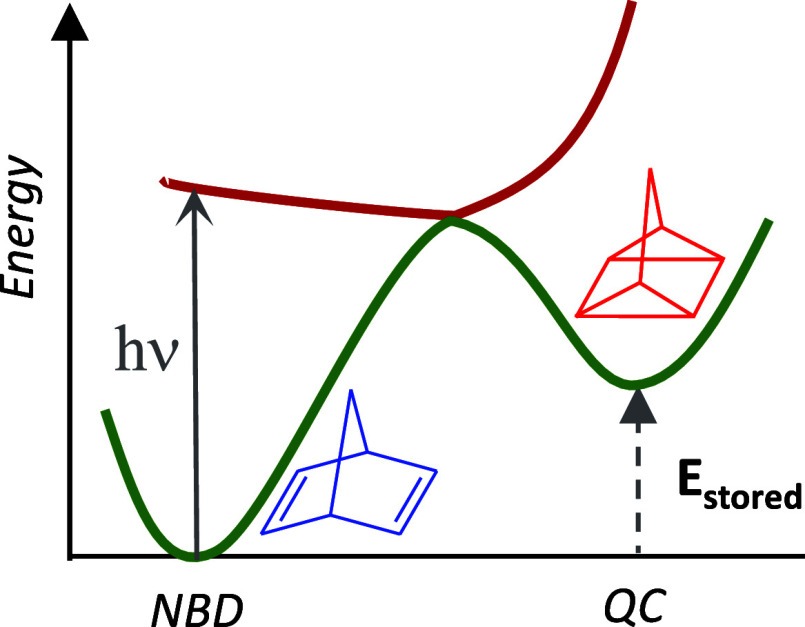
Schematic potential energy surfaces (ground state and first excited state) for the NBD/QC system. The stored energy (*E*_Stored_) represents a fraction of the absorbed photon energy and can be released back in a controlled manner to recover the NBD system and generate heat.

In the closed configuration, QC, the system acts as an energy storage reservoir, which is reached from the open configuration (NBD) by absorbing solar energy and undergoing photochemical cyclization. This transformation is initiated by photon absorption in the visible range, triggering a reversible ring-closing reaction. The stored energy in the QC form can be retained until needed, and upon demand, the system can undergo a controlled thermal or photochemical back-reaction, converting QC back to NBD and releasing the stored energy in the form of heat (see Figure 1).^17^

Key aspects of the NBD/QC MOST system include its tunable absorption properties, allowing efficient utilization of solar radiation and the ability to modulate the release of stored energy through controlled isomerization processes. Understanding and optimizing the chemical properties and photochemical processes of the NBD/QC system is crucial for enhancing its overall performance in solar thermal applications.^10^ The exploration of substituent effects and molecular design principles play a pivotal role in tailoring these systems for practical and efficient solar energy utilization.^18^

In this study, we present a systematic approach to investigate the impact of substituents in polysubstituted compounds on various chemical or physical properties. Our methodology is applied to the design of molecular solar thermal (MOST) systems, focusing on the crucial aspect of energy storage. We found that by utilizing information from a small subset of the compound library we can accurately estimate properties for the remaining systems. This allows for predictions within large compound libraries at relatively low computational costs. Furthermore, our results indicate a correlation between this predictive approach and gradual forecasting of the electronic density of the target compounds. This correlation provides insight into the substituent effects, enhancing our understanding of the molecular design principles governing the MOST systems.

## Methodology

Density functional theory (DFT) methodology has been used, specifically the M06-2X hybrid functional,^19^ to perform electronic structure calculations that have been proved to render good results for NBD/QC reaction energies.^20^ All calculations were carried out using the 6-311++G(d,p) basis set. All of the electronic structure calculations were performed using Gaussian16 suite of programs.^21^

To generate the structure of the 351 derivatives, an own developed code was used to systematically generate initial structures of both their NDB and QC forms. These structures were subsequently optimized at the same level of theory. To ensure that these structures correspond to true minima on the potential energy surface, we verified the optimization results using the approximate Hessian obtained during the optimization process.

Electron density calculations were conducted using the same DFT methodology (M06-2*X*/6-311++G(d,p)). Additionally, electron density differences were computed using our own custom-developed code using electron density cubes generated by Gaussian.^21^

## Results

Given a chemical compound with different substitution positions and considering a set of possible substituents, it is possible to generate a very large number of different derivatives forming a chemical library. In order to make predictions of a large set of compounds obtained from chemical substitutions, a perturbation-like methodology has been developed, where only information from a small subset of the chemical library is necessary to make predictions on the entire set of possible compounds. This approach is described here, showing the different orders of approach to the problem for predicting a given molecular property. Additionally, the perturbational-like approach is applied to the progressive construction of the electronic density function within different orders of approach. This methodology is then applied to the study of stored energy in a molecular solar thermal system (NBD/QC).

### Methodology for Systematic Inclusion of Substituent Effect

In the framework of combinatorial chemistry, the construction of a compound library involves introducing substituents at various sites of a reference molecule in a systematic way. When one focuses on a specific physical or chemical property of the system, the challenge arises in predicting or determining this property for the entire compound library. In this context, we propose a systematic approach for predicting any property of a molecular system across the entire library by leveraging information obtained from a chosen subset of compounds. This approach accounts for the successive effects of substitution on the target property, treating the effect of a polysubstituted compound initially as the sum of the individual effects of monosubstituted derivatives. Second- and higher-order corrections are introduced to refine these predictions. In the subsequent sections, we present the details of the predictive model and apply this systematic approach to predict properties in polysubstituted Norbornadiene/Quadricyclane (NBD/QC) systems, with a particular focus on the reaction energy governing their interconversion.

**Figure 2:**
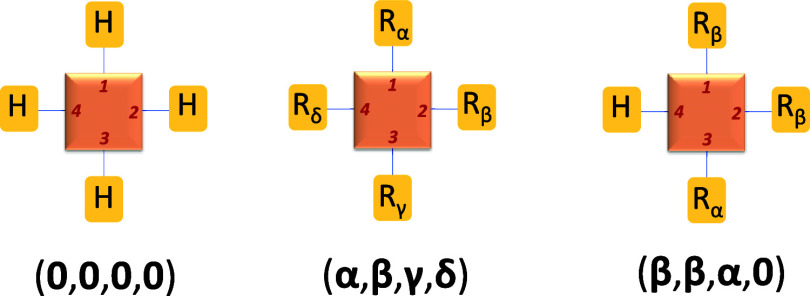
Examples of the nomenclature used to denote a polysubstituted compound in a four-position compound. The unsubstituted (–H) compound is denoted as (**0**, **0**, **0**, **0**). Another tetra-substituted compound with substituents *R*_α_, *R*_β_, *R*_γ_, and *R*_δ_ in positions 1 to 4, respectively, is denoted as (**α**, **β**, **γ**, **δ**), and a trisubstituted compound with *R*_β_, *R*_β_, *R*_α_, and H in positions 1–4, respectively, is denoted as (**β**, **β**, **α**, **0**).

Regarding the nomenclature used in the following, a polysubstituted molecular system can be specified by an *n*-tuple, such as (***α***, ***β***, ***γ***, ···), indicating the presence of a substituent ″***α***″ at position ″1,″ ″***β***″ at position ″2,″ and so on. Conversely, the unsubstituted system, characterized by hydrogens at all substitution positions, is denoted by the *n*-tuple (**0**, **0**, **0**, ···, **0**) (see Figure 2). Our objective is to predict a molecular property, denoted as Π, for a specific member within the chemical library. Thus, the value of this property for the polysubstituted system is expressed as Π(**α**, **β**, **γ**, ···), while for the unsubstituted system, it is denoted as Π(**0**, **0**, **0**, ···). This latter value is the reference value for the property Π and will be denoted for simplicity as Π(**0**). In the following, different approaches are presented in order to predict this property in the polysubstituted compound.

#### First-Order Approach

Before going ahead with the definition of the first-order approach, let us consider a monosubstituted system, where the *i*th position is substituted with **α**. The system is represented by a tuple (**0**, ···, **α**, ···, **0**). The value of the property Π for this system is defined as
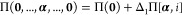
1where Δ_1_Π[**α**,*i*] defines the variation of the property Π induced by the substituent **α** in the position *i*th position. Therefore, Δ_1_Π[**α**,*i*]exactly quantifies the variation on the property Π due to the monosubstitution of the system (in the *i*th position with the substituent **α**).

The first-order approach in the prediction of a Π property in a polysubstituted molecular system can be defined as
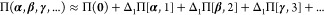
2

Or equivalently:
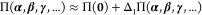
3where Δ_1_Π(**α**, **β**, **γ**, ···) stands for the first-order correction of the property Π and Π(**0**) is the zeroth-order approach (i.e., the property value for the unsubstituted system).

Equation 3 can be interpreted in the following terms: the effect of poly substitution in a given property of the molecular system can be, in a first approach, determined by the sum of individual effects of each monosubstitution. In this way, for instance, the effect on the reaction energy of different chemical substitutions is the reaction energy of the unsubstituted system plus the variation due to each single monosubstitution.

#### Second-Order Approach and Further Corrections

Before defining additional corrections, let us investigate the value of the property Π in a disubstituted system with substituents **α** and **β** at positions *i* and *j*, respectively. This value is exactly:
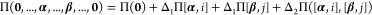
4

Equation 4 introduces the definition of Δ_2_Π([**α**, *i*],**[β**, *j*]), which corresponds to the second-order contribution to the property Π in a disubstituted system with substituents **α** and **β** at positions *i* and *j*, respectively. Δ_2_Π([**α**, *i*],[**β**, *j*]) quantifies the variation of the property Π in a disubstituted system where position *i* is substituted by **α** given that position *j* is substituted by **β**. This magnitude corresponds therefore to the cooperative effect in the property Π due to the simultaneous presence of both substituents.

Following the same approach given by eqs 2 and 3, a second-order correction of the property Π can be defined. Again, let us consider a polysubstituted system (**α**, **β**, **γ**, ···, **ω**). The molecular property Π can be approximated as
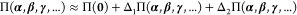
5where Δ_2_Π(**α**, **β**, **γ**, ···) stands for the second-order correction to the property Π in a polysubstituted system and is defined as
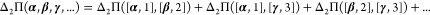
6where the right-hand side of eq 6 runs over all of the possible pairs of substituents that can be considered in the polysubstituted system. The summation of all of these terms conforms the second-order correction to the property Π in a polysubstituted system (**α**, **β**, **γ**, ···), i.e., Δ_2_Π(**α**, **β**, **γ**, ···).

Analogously, higher-order corrections can be defined. The number of possible corrections in a given system is exactly the number of substitution positions in this system. By definition, if all possible corrections are included, the value of the Π property is exactly determined. Consequently, for a system with “*N*” substitution positions:
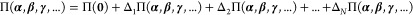
7where it should be remarked that in eq 7 the right-hand side equals exactly the left-hand side, opposite to eqs 3 and 5 where we have only an approximation to the Π(**α**, **β**, **γ**, ···) property.

#### Substituent Effect in Terms of Electronic Density Variation

The treatment described above is applicable for any molecular property Π, no matter if it is a scalar, vector, tensor, ··· or even a function. Therefore, it can be implemented for the electronic density. The electronic density can be constructed from successive corrections using eq 7:
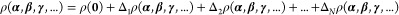
8

where *N* is the number of positions available to substitute. The two first variations are especially relevant for understanding the effect of the substitution in the electron density. The first-order variation of this property due to the presence of a substituent **α** at the *i*th position is
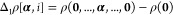
9

which is just the difference between the monosubstituted compound’s electronic density and that of the unsubstituted compound. Δ_1_ρ[**α**, *i*] is therefore the individual effect of a substituent **α** at the *i*th position in the total electronic density.

The second-order variation of the electronic density due to the inclusion of substituents **α** and **β** at positions *i* and *j*, respectively, is therefore
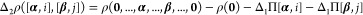
10

Δ_2_ρ([**α**, *i*],[**β**, *j*]) provides the variation in the electronic density due to the cooperative effect of substituents **α** and **β** at positions *i* and *j*, respectively.

#### Computational Efficiency of the Approach

**Figure 3:**
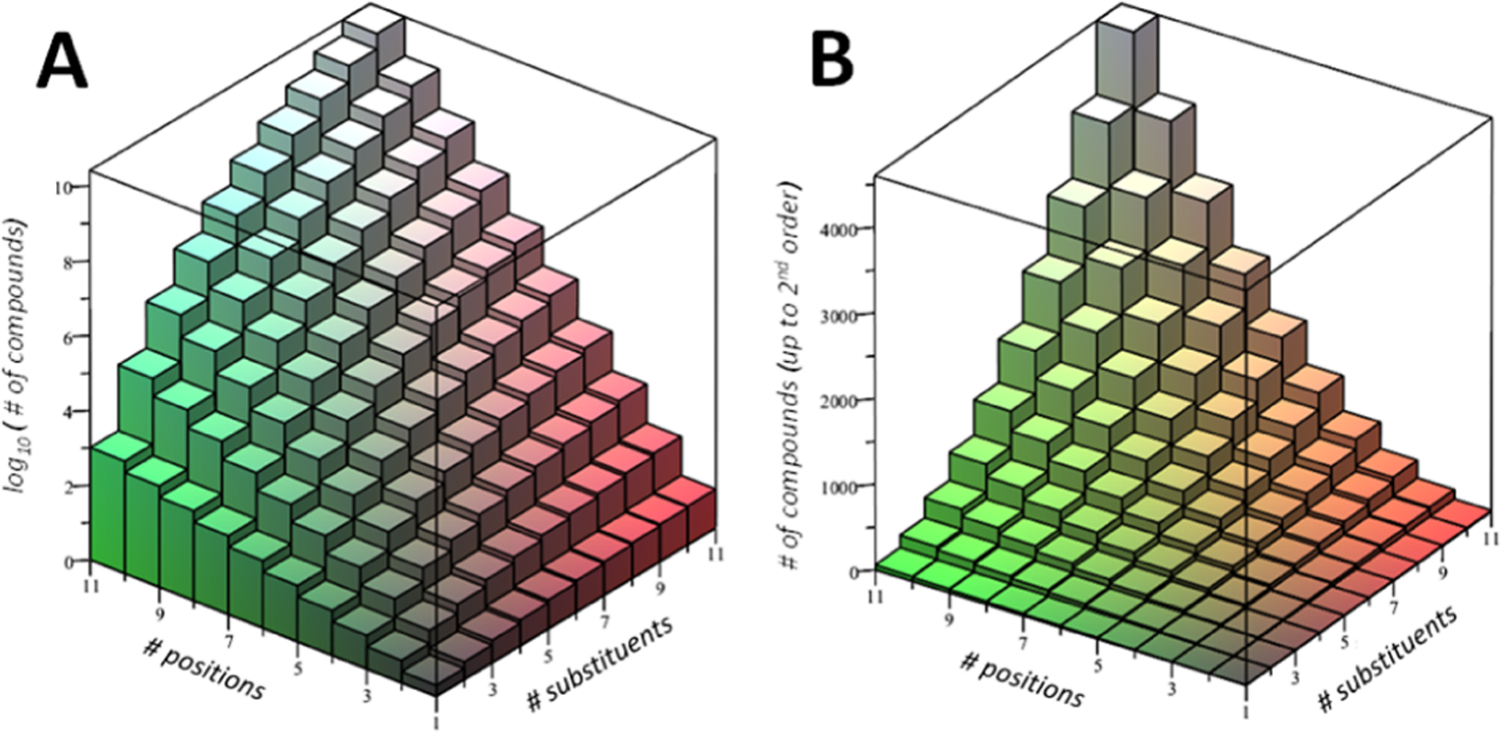
(A) Presentation of the log_10_ of the total number of possible compounds within a library (i.e., from 1 to 10 substituents and positions of substitution), which is given by *M* log_10_(*N* + 1). (B) Number of compounds necessary to reach the second-order correction as a function of the number of substituents and positions (i.e., from 1 to 10).

Approaching a molecular property of a polysubstituted compound using up to first- or second-order approach as defined in eqs 3 and 5 may imply saving a very large amount of computational time. It depends on the number of positions that can be substituted, the number of substituents defining the library, and potential symmetry constraints. Nevertheless, for a given chemical library, the fraction of compounds necessary to compute to make predictions for the whole library rapidly drops with an increasing number of substituents and positions of substitution. For instance, if we consider 8 possible substitution positions and 5 substituents, in order to reach a second-order approach for a given property, it is necessary to compute 741 molecules of a total of 1.68 × 10^6^ possible compounds, which means determining only the 0.044% of the whole library. For 8 substituents and 8 possible substitution positions, the number of compounds of the library is ca. 10^8^, while second-order correction is reached by computing only 2341 compounds (0.0023% of the whole library), saving very large computational time. Different cases (i.e., from 1 to 10 substituents and positions of substitution) are represented in Figure 3. As can be inferred, only a small fraction of the total number of possible compounds needs to be computed, and this fraction decreases with increasing library complexity.

In general, if *N* is the number of substituents and *M* is the number of positions, the number of possible compounds is (*N* + 1)^*M*^, while the number of monosubstituted and disubstituted compounds is *N*·*M* and , respectively. Therefore, the fraction of the compounds to be computed to reach a second-order approach is
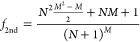
11

Basically, the numerator increases quadratically with *N* and *M*, while the denominator increases exponentially as *N*^*M*^, making the fraction of compounds to be computed within the second-order approach decrease exponentially as *N* and *M* increase.

### The NBD/QC Derivatives Data Set

In order to explore the improved performance of the NBD/QC MOST system, it is possible to explore a combinatorial chemistry approach, where a set of substituents generates a library of compounds. This approach generates a large number of molecular systems in a systematic way where the number of them depends on the number of substituents, the number of substitution sites, and eventually symmetry constraints.

**Figure 4:**
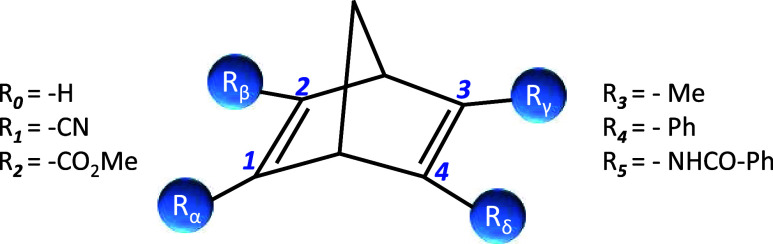
Structure of NBD where the positions for substitution are indicated: carbon atoms 1, 2, 3, and 4. A substituent is linked to each position: *R*_α_, *R*_β_, *R*_γ_, and *R*_δ_, respectively. Therefore, this compound is denoted by (**α**, **β**, **γ**, **δ**).

In the case of the NBD/QC system, a total of 4 substitution positions and 5 substituents have been considered to implement the developed methodology (see Figure 4). In the following, we will use a 4-tuple to indicate the specific compound following the numbering: **0** = –H, **1** = –CN, **2** = –CO_2_Me, **3** = –Me, **4** = Ph, **5** = NHCO-Ph. The first position of the 4-tuple indicates the substituent on carbon 1 and so forth for the rest of the positions (see Figure 4).

This set of substituents yields a total of 1296 compounds if symmetry constraints are ignored. By considering Burnside’s lemma, it is possible to determine the number of distinct compounds (combinatorial problem) considering the restrictions imposed by the system’s symmetry constraints (i.e., they may belong to point group *C*_2*v*_, or subgroups *C*_2_ or *C*_S_). When symmetry restrictions are considered, a total of 351 different compounds are possible.

In this case study, the NBD/QC derivatives within the proposed library are studied as potential MOST systems. More specifically, we focus on the prediction of the storage energy of the compounds (*E*_stored_, see Figure 1), which will be the property Π to be predicted using the developed formalism.

### Prediction of Storage Energy in NBD/QC Substituted System

In the following, we will apply the proposed approach to the study of the storage energy in the NBD/QC system. Therefore, the molecular property to consider is the reaction energy or stored energy (*E*_stored_, see Figure 1), which for simplicity will be denoted as *E*. This corresponds to a fraction of the absorbed photon energy stored as chemical energy in the system.

The library of compounds is confirmed, as described above, by a total of 5 substituents (including, for simplicity, –H in the definition): **0** = –H, **1** = –CN, **2** = –CO2Me, **3** = –Me, **4** = –Ph, **5** = –NHCO-Ph, and 4 different substitution positions (see Figure 4). A total of 351 compounds are possible once the symmetry constraints are considered through Burnside’s lemma. There is a reference compound (**0**, **0**, **0**, **0**), and 5 different monosubstituted compounds, e.g. (**3**, **0**, **0**, **0**) is the methyl-substituted compound, which is equivalent to (**0**, **3**, **0**, **0**), (**0**, **0**, **3**, **0**) and (**0**, **0**, **0**, **3**). There are 45 different disubstituted compounds, 125 trisubstituted compounds, and 175 tetrasubstituted compounds, i.e., 351 possible compounds including the reference system.

The Δ_1_*E* [**α, i**], i.e., the first-order variation of the storage energy due to the monosubstitution with **α** substituent at position *i* (see eq 1), quantities are listed in Table 1. All of the first-order contributions to energy are positive except for substituent **2**.

**Table 1:**
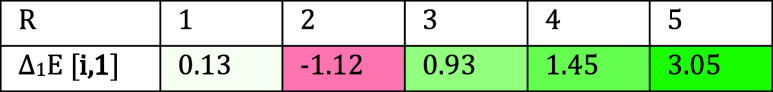
First-Order Variation of the Reaction Energy for NDB to QC Isomerization for Single-Substituted Compounds^a^

a Energies are indicated in kcal/mol with green and red color scale.

**Figure 5:**
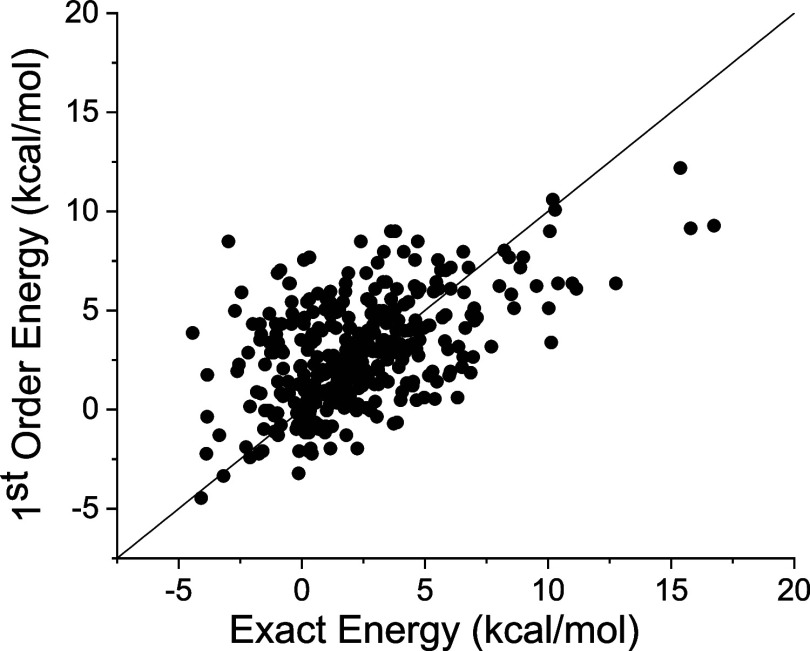
First-order approach energies were computed by using eq 2. The predicted data for 345 compounds is based on the calculation of just 5 compounds and the corresponding reference (unsubstituted) compounds.

By computing only 5 compounds along with the reference (unsubstituted) compound, the rest of the library compounds, i.e., 345 compounds, can be predicted by using eq 2. The results are summarized in Figure 5. As can be seen, the correlation between predicted energy using the first-order approach and the computed energy is apparent. The standard deviation of the predicted energies is 3.0 kcal/mol, which indicates a qualitative correlation between the exact and predicted energies. Additionally, most of the predicted energy variations are positive, since all values of Δ_1_*E* are positive but one.

The exact second-order correction can be computed for all possible disubstituted compounds by using eq 4. For the present case, a total of 45 systems have to be computationally determined; i.e., their isomerization reaction energies have to be computed. Therefore, from 51 calculations of the stored energy, a total of 300 additional compounds can be predicted by using eq 5. The 45 computed second-order corrections to the reaction energy are indicated in Table 2.

**Table 2:**
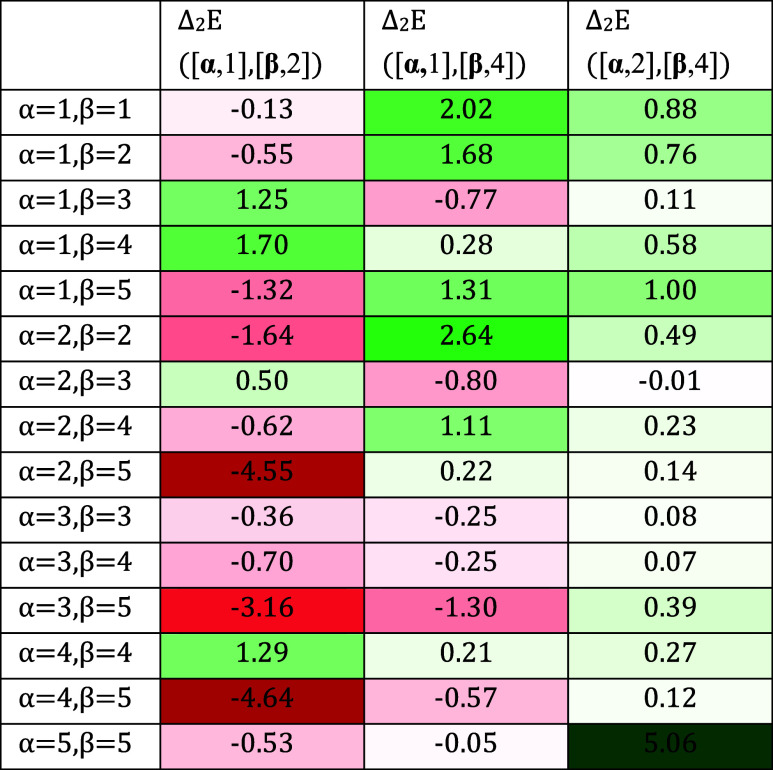
Second-Order Variation of the Reaction Energy for NDB to QC Isomerization for Double-Substituted Compounds (45 Possible Disubstituted Compounds)^a^1

a The first column, ([**α**, 1],[**β**, 2]), indicates disubstituted compounds at positions 1 and 2, while second ([**α**, 1],[**β**, 4]) and third ([**α**, 2],[**β**, 4]) columns correspond to 1-4 and 2-4 disubstituted compounds, respectively. Energies are indicated in kcal/mol with green and red color scales as in Table 1.

It must be noted that second-order corrections to energy are generally smaller than first-order corrections, but in some cases, they are of comparable magnitude. Additionally, the complex interplay between pairs of substituents makes this energy take both positive and negative values.

**Figure 6:**
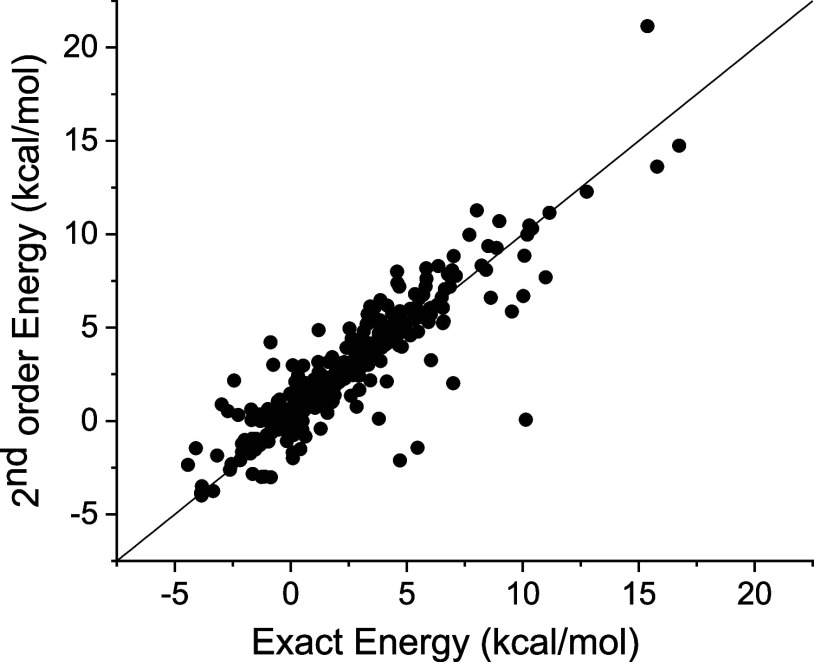
Second-order approach energies predicted using eq 4. The predicted data for 300 compounds is based on the calculation of 50 compounds (mono- and disubstituted) and the corresponding reference (unsubstituted) compounds.

With this information, the NBD to QC reaction energies can be predicted up to the second-order approach. The error is significantly decreased in comparison to first-order approach, as the standard deviation of the predicted energies drop up to 1.5 kcal/mol (see Figure 6).

Only 17 compounds (out of 300 predicted) have an error energy larger than two times the standard deviation, while only 8 have an error larger than 3 times the standard deviation. This means that the reaction energy of 283 compounds is predicted with an error smaller than two times the standard deviation (i.e., 2.93 kca·mol^–1^). In this study, the selected substituents exhibit limited conformational flexibility, ensuring that geometry optimizations yield the minimum energy structures. However, for systems with more flexible substituents, conformational sampling would be necessary to account for potential low-energy conformers. This could be addressed in future applications by incorporating systematic conformational searches or molecular dynamics to improve the robustness of the methodology proposed here.

Regarding the consistency of different corrections, it has to be noted that each approach implies decreasing corrections to stored energy. On average, the first-order correction (calculated as the root-mean-square of the energy variation) represents 46.1% of the energy variation due to substitution, while the second-order correction implies 32.8% of the total energy variation, and third- and fourth-order corrections together sum a 21.1% of the total energy variation. This confirms the intuitive assumption that the increasing corrections has less impact on the predicted energies, as it is apparent from Figures 5 and 6.

Of course, out of the 300 compounds predicted using the second-order approximation, there are compounds that exhibit significant error (8 of them have an error greater than 4.4 kcal/mol). In all of these cases, the reason for the deviation is the same: cooperative interactions of three or four substituents are significant. This phenomenon can be easily understood as follows: two substituents exhibit certain interactions that are altered in the presence of a third or fourth substituent. Therefore, the second-order approximation, which accounts for interactions only between pairs of substituents, is not entirely accurate.

**Figure 7:**
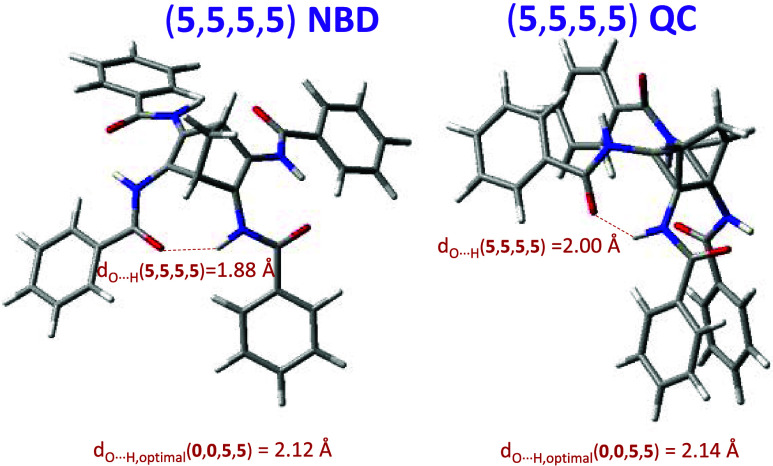
(**5**, **5**, **5**, **5**) NBD and QC structures. The highly strained chain of hydrogen bonds in NBD makes the system have higher energy than predicted from the relaxed two-substituent compound (**0**, **0**, **5**, **5**). The O···H distance in crowded (**5**, **5**, **5**, **5**) NBD is 1.88 Å, while for the relaxed (**0**, **0**, **5**, **5**) NBD, it is 2.12 Å. The differences between (**5**, **5**, **5**, **5**) and (**0**, **0**, **5**, **5**) in QC are much lower, with the energy prediction being overestimated.

To exemplify this phenomenon, we will focus on the compound (**5**, **5**, **5**, **5**), which is the system with the highest predicted storage energy (using a second-order approximation) and one of the systems with the largest error. This compound has a reaction energy variation of 15.38 kcal/mol. However, the energy variation predicted based on the second-order approximation is 21.14 kcal/mol. In this case, the energy is overestimated mainly due to the stability of the NBD, which is smaller than predicted using the second-order approach. The reason is that the interactions between the substituents of type ″5″ involve the formation of hydrogen bonds. These hydrogen bonds are formed optimally when there are only two substituents in the system, with the N–H···O distance equal to 2.12 Å in the case of the compound (**0**, **0**, **5**, **5**). But, when having four bulky substituents together (**5**, **5**, **5**, **5**), the system becomes significantly constrained due to steric repulsions, but the optimal structure maintains the formation of all the hydrogen bonds (see Figure 7), albeit weakened (i.e., N–H···O distance equal to 1.88 Å), which leads to an increase in the energy (destabilization) of the NBD and consequently reduces the stored energy of the real system.

This effect is less pronounced in the QC, where the orientation of the substituents ensures that the presence of a third or fourth ″5″ group does not excessively affect the interaction between two of them (see Figure 7). Thus, the prediction using the second-order approximation overestimates the stored energy by approximately 6 kcal/mol.

**Figure 8:**
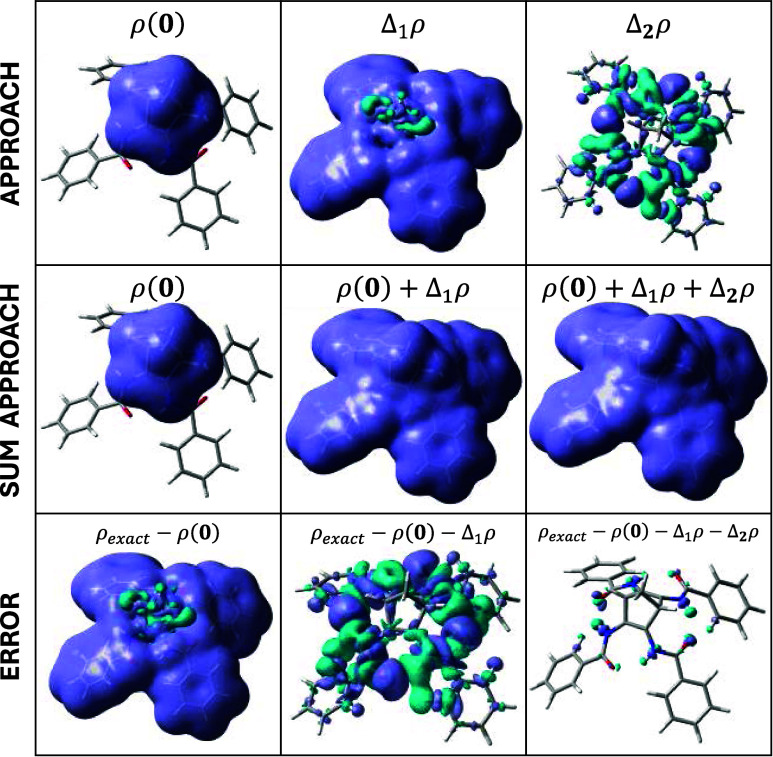
Electronic density isosurfaces corresponding to 0.004a.u. isovalues for the (**5**, **5**, **5**, **5**) NBD compound (i.e., –PhOMe at all positions). (Top) Different approaches to the total electronic density of the compound: zeroth-, first-, and second-order approaches: ρ(**0**), Δ_1_ρ and Δ_2_ρ. (Middle) Addition of the different approaches and (bottom) the difference (error) with respect to the exact electron density. Positive isosurfaces of Δρ (and ρ, which is inherently always positive) are displayed in violet, while the negative isosurfaces of Δρ are shown in cyan.

A very interesting property to analyze with the proposed methodology is the electronic density with substitution. Let us consider again the (**5**, **5**, **5**, **5**) NBD compound (i.e., –NHCO-Ph at all positions), which is the predicted compound with the largest storage energy and one presenting the largest errors in the prediction. By determining just the electronic density of the unsubstituted compound (**0**, **0**, **0**, **0**) and the 4 monosubstituted systems, i.e., (**5**, **0**, **0**, **0**), (**0**, **5**, **0**, **0**), (**0**, **0**, **5**, **0**), and (**0**, **0**, **0**, **5**), an approximated electronic density of the target compound is obtained. This electronic density is the summation of the single first-order corrections for each substituent (according to eq 9), therefore this correction equals: Δ_1_ρ(**5**, **5**, **5**, **5**) = Δ1ρ[**5**, 1] + Δ_1_ρ[**5**, 2] + Δ_1_ρ[**5**, 3] + Δ_1_ρ[**5**, 4]. The first-order approximation for the electronic density is given by ρ(**0**) + Δ_1_ρ(**5**, **5**, **5**, **5**). The second-order approximation further refines the result as ρ(**0**) + Δ_1_ρ(**5**, **5**, **5**, **5**) + Δ_2_ρ(**5**, **5**, **5**, **5**) (see Figure 8).

The exact electronic density ρ_exact_ = ρ(**5**, **5**, **5**, **5**) and the first-order (ρ(**0**) + Δ_1_ρ) and second-order (ρ(**0**) + Δ_1_ρ + Δ_2_ρ) approximations exhibit relatively small differences. It is possible to define the electronic density error function: for instance, the second-order electronic density error (Figure 8) is given by eq 12.
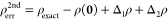
12

Integrating the absolute value of the error function (therefore avoiding cancelation of errors due to the sign of the function) for each approximation, it is possible to quantify the difference between exact and approximated electronic density functions in terms of the number of electrons. The zeroth-order approach integrates to ca. 256e (corresponding to neglecting the four substituents). The first-order approach error function integrates to 1.25e, while the second-order approach integrates only to 0.13e, showing the small differences between the exact electronic density and the approximations.

The larger components of the ρ_err_^1st^ function are mainly located in the intersections between substituents, bringing to the forefront the fact that this approach neglects the interaction between substituents. Similarly, ρ_err_^2nd^ has relevant values in the intersection between substituents since it mainly quantifies the variation in the interaction between pairs of substituents.

As has been demonstrated elsewhere,^4^ the electronic density is related to the forces induced by the substituents, which is directly related to the variation in the reaction (or activation) energy. Nevertheless, applying such analysis of electronic density variations is out of the scope of the present investigation, although it could provide a rational explanation to the link between electronic properties of polysubstituted systems and the variation of the electronic density function.

## Conclusions

Here, we propose a systematic approach to predict a given chemical or physical property in a polysubstituted compound based on a library of substituents. Successive approximations, including first-order and higher-order corrections, can be systematically incorporated. This protocol may permit the predictions of these properties for a large number of derivatives using data from only a small subset of compounds.

In the case study, a library of NBD/QC derivatives is studied. In order to maximize the storage energy in this MOST system, the proposed systematic approach has been applied, showing that knowing just 6 reaction energies (i.e., one corresponding to the unsubstituted system and 5 corresponding to monosubstituted compounds) it is possible to qualitatively predict 345 unknown compounds within only a 3.0 kcal/mol standard error. This means that it is only necessary to determine the properties of less than 2% of the total library of compounds. Second-order corrections using the information from 51 compounds provide significantly accurate predictions with a standard deviation of only 1.5 kcal/mol.

The systematic approach presented here can be employed to any combinatorial chemistry problem and may be useful in screening tasks when looking for optimal chemical or physical properties using the substitution strategy. This approach saves important computational time since only a small fraction of the total library of compounds has to be characterized, computationally or experimentally, to generate good predictions for the whole library.

## Supporting Information Available

Stored energies of each compound of the library (XLSX)

## Dedication

This work is dedicated to Prof. Obis Castaño on the occasion of his 81st birthday.
